# Memory precision for salient distractors decreases with learned suppression

**DOI:** 10.3758/s13423-021-01968-z

**Published:** 2021-07-28

**Authors:** Bo-Yeong Won, Aditi Venkatesh, Phillip P. Witkowski, Timothy Banh, Joy J. Geng

**Affiliations:** 1 Center for Mind and Brain, University of California Davis, Davis, CA USA; 2 Department of Psychology, University of California Davis, Davis, CA USA; 3 Department of Radiology, University of California San Francisco, San Francisco, CA USA

**Keywords:** attention and memory, attention capture, visual working memory, distractor suppression

## Abstract

Attention operates as a cognitive gate that selects sensory information for entry into memory and awareness (Driver, 2001, *British Journal of Psychology, 92*, 53–78). Under many circumstances, the selected information is task-relevant and important to remember, but sometimes perceptually salient nontarget objects will capture attention and enter into awareness despite their irrelevance (Adams & Gaspelin, 2020, *Attention, Perception, & Psychophysics, 82*[4], 1586–1598). Recent studies have shown that repeated exposures with salient distractor will diminish their ability to capture attention, but the relationship between suppression and later cognitive processes such as memory and awareness remains unclear. If learned attentional suppression (indicated by reduced capture costs) occurs at the sensory level and prevents readout to other cognitive processes, one would expect memory and awareness to dimmish commensurate with improved suppression. Here, we test this hypothesis by measuring memory precision and awareness of salient nontargets over repeated exposures as capture costs decreased. Our results show that stronger learned suppression is accompanied by reductions in memory precision and confidence in having seen a color singleton at all, suggesting that such suppression operates at the sensory level to prevent further processing of the distractor object.

**Supplementary Information:**

The online version contains supplementary material available at 10.3758/s13423-021-01968-z.

## Introduction

In order to accomplish goal-oriented behaviors, it is necessary to suppress distractions. Failures to do so will delay task completion or may even derail it completely. While it is clear that distractor suppression is integral to goal-oriented behaviors, how this is accomplished and how this affects downstream cognitive processes is still poorly understood. All models of attention posit that attention operates as a gating mechanism in which selected objects are processed more deeply and have a greater likelihood of entry into memory and awareness (Driver, 2001). In these studies, we test the attentional gating hypothesis for distractor suppression and ask whether memory and awareness for salient distractors are related to the initial strength of attentional capture (or strength of suppression) over time.

The bulk of research on attentional mechanisms has focused on target selection, and it has been debated whether suppression occurs outside of target selection. It is becoming increasingly clear, however, that suppression of task-irrelevant information can occur independently from target selection (Chang & Egeth, 2019) and is likely supported by multiple cognitive mechanisms (Chelazzi et al., 2019; Fang et al., 2019; Gaspelin & Luck, 2018b; Geng et al., 2019; Moher & Egeth, 2012; Noonan et al., 2018). A number of these studies have found that suppression of both spatial and nonspatial distractor features is particularly effective when properties of task-irrelevant stimuli are predictable (Chetverikov et al., 2017; Ferrante et al., 2018; Gaspelin & Luck, 2018a; Geng & Diquattro, 2010; Geyer et al., 2006; Stilwell & Vecera, 2019; van Moorselaar & Slagter, 2019; Vatterott et al., 2018; Vatterott & Vecera, 2012; Wang & Theeuwes, 2018). For example, Vatterott and Vecera (2012) found that distractor interference decreased when a salient color singleton distractor appeared repeatedly, but attentional capture returned when the color changed. This rebound did not occur, however, when the color of the singleton distractor was expected to vary (Vatterott et al., 2018; Won et al., 2019). These results suggest remarkable flexibility in using feature-specific properties or abstract rules to actively suppress distractors.

Although task-irrelevant, but salient, stimuli are more likely to capture attention, the relationship between involuntary attentional capture and subsequent attentional engagement, memory, and awareness is still debated (Zivony & Lamy, 2016, 2018). Two recent studies, however, have shown a relationship between perceptual salience, attention, and awareness (Adams & Gaspelin, 2020; Constant & Liesefeld, 2021). Constant and Liesefeld (2021) used a parametric manipulation of salience and found a monotonic relationship between bottom-up saliency and the probability of memory for the item, although interestingly, the precision of the memory was not affected by saliency. Relatedly, Adams and Gaspelin (2020) found that participants were more likely to report awareness of a color singleton distractor from visual search trials with larger RT capture effects (Belopolsky et al., 2008). These results lead to the prediction that the opposite should also be true: Memory precision and awareness should decline as suppression improves. Such a result would be consistent with recent findings that reductions in distractor interference are related to mechanisms within visual cortex that prevent the “readout” of attentional priority signals to later stages of processing (Adam & Serences, 2020; Birman & Gardner, 2019; Won et al., 2020).

The purpose of the current studies was to determine whether learned suppression of salient distractors during visual search produces a concomitant reduction in subsequent memory probability and precision for the salient distractor. Measuring the outcome of distractor processing can be difficult because it requires participants to report on a feature that they are supposed to ignore. Previous studies circumvented this issue by measuring residual spatial attention as a proxy for which objects were attended (Gaspelin et al., 2015; Kim & Cave, 1995; Won et al., 2019). While this is an elegant method, it does not provide a direct measurement of attention to specific stimulus features. In the present study, we directly measure the consequence of attentional suppression of color singleton distractors as a function of repeated exposure. We do so by harnessing large numbers of participants engaging in a one-trial memory probe following a varying number of trials of color singleton distractors (Mack & Rock, 1998; Won et al., 2019). Our results provide direct evidence for a relationship between learned distractor suppression and memory and awareness of the distractor.

## Experiment 1A

### Method

#### Participants

Four hundred twenty-eight subjects recruited from UC Davis participated in an online experiment (testable.org) for course credit through SONA. One hundred eight subjects (25.2% of subjects) were excluded from analyses due to performance lower than 80% accuracy on the visual search trials. The high number of individuals with poor performance is likely due to the fact that this was an online experiment administered through SONA and course credit was not tied to performance. This resulted in the inclusion of 320 participants (mean age = 20.6, *SD* = 2.4, female = 219, male = 99, other = 2, left-handed = 30, right-handed = 290). Forty participants were randomly assigned to each of eight groups. Each group consisted of two subgroups to counterbalance the two *critical singleton* colors, but no differences in color were expected, and the data were collapsed to increase statistical power. The sample size of *N* = 20 for each subgroup was determined based on a previous study from which we adopted the experimental design (Won et al., 2019). A power analyses was conducted based on differences between group means in RT for the high frequency singleton and the low frequency singleton conditions in Experiment 1 of Won et al. (2019), which had an effect size of *d* = 1.2, and an alpha of .05. Results showed that a sample of 16 participants was required to achieve a power of .80. Our final sample size of 20 participants per subgroup (40 per condition of interest after collapsing between *critical singleton* colors, which was expected to be a manipulation of noninterest) was used to buffer against additional noise expected from online experiments and the single-trial approach. All participants had normal or corrected-to-normal vision and provided informed consent in accordance with NIH guidelines provided through the UCD Institutional Review Board.

#### Stimuli and apparatus

**Fig. 1:**
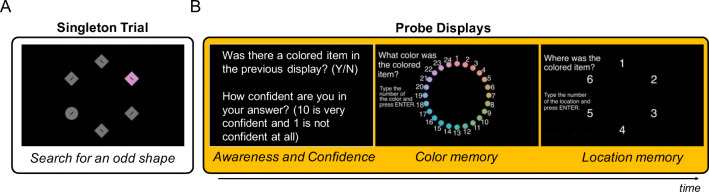
Search and probe displays. **a** An example of a singleton-present trial. Participants were asked to find the odd shape and indicate the orientation of the bar inside with a manual response. In this example, the target is the circle, and the bar is tilted to the right. **b** After a fixed number of trials (determined by the *critical singleton group*), participants encountered a set of surprise probe displays that assessed memory and awareness of the preceding color singleton

Search displays: Search displays were generated from MATLAB 2019a (The MathWorks, Natick, MA, USA). Search displays contained six shapes drawn in gray, five diamonds, (1.7° × 1.7°) and one circle (1.5° in diameter) or one diamond and five circles on a black background. The eccentricity (center of each item to the center of the screen) was 4° based on an estimated viewing distance of 60 cm. Each shape contained a black line subtending 0.1° × 0.5° that was randomly tilted 45° to the left or right (see Fig. 1a). Note that the stimulus size and color might have varied depending on the participant’s environment (e.g., monitor, video card, screen specifications, and lighting conditions). The target was defined by the “odd” shape (i.e., a circle among diamonds or a diamond among circles). This paradigm is known to induce singleton detection mode, which should increase attentional capture by the singleton distractor (Bacon & Egeth, 1994; Theeuwes, 1992). The target appeared equally often in six positions and was always gray. The other five positions were occupied by a nontarget. On 6 out of the total 30 trials, all nontargets were an identical gray color to the target. On 24 out of 30 trials, one distractor was a unique color. The singleton color was different on each trial it was present; the colors were equidistant and selected from CIE Lab color wheel (radius: 39, luminance: 70, a = 0, b = 0). The critical singleton was always one of two colors, counterbalanced across participants: RGB for the pink color = [218; 148; 208] and RGB for the green color = [110; 187; 134]. Two colors were used to ensure that our results were not due to spurious effects associated with one color and to minimize color variation due to variability in monitor settings and differences in color perception.

Probe displays: The set of probe displays consisted of four questions on three consecutive displays. In the first display, two questions were shown, “Was there a colored item in the previous display (Yes or No)?” and “How confident are you in your answer (10 is very confident and 1 is not confident at all)?” Regardless of their answers, a second display containing the question, “What color was the colored item?” and a color wheel that consisted of 24 colors (15 degrees apart) was shown. Finally, a location probe appeared with the question, “Where was the colored item?” with six alternative location choices (see Fig. 1b). The color probes were of primary interest given the singleton was defined by color saliency. The location probes were included for exploratory analyses. The location probe display always came last and were temporally distant from the actual search display. We therefore expected the results to be noisier than expected for a targeted working memory assessment of location. For these reasons, the data are only reported in the Supplemental Materials (S4).

#### Design and procedure

**Fig. 2:**
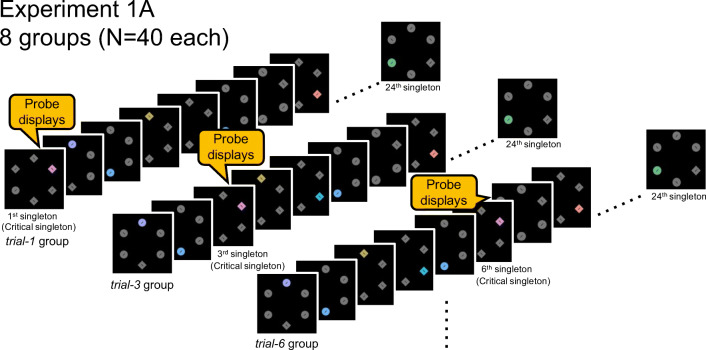
Trial procedure in Experiment 1A. Each group consisted of 40 participants. Participants in all eight groups saw the same visual search and probe displays. The only difference between them was the trial position of the surprise set of memory and awareness probe displays. The singleton-present trial immediately preceding the probe displays is referred to as the *critical singleton trial*

We collected data through Testable (testable.org), an online experiment platform. The experiment began with instructions and four practice trials followed by the main experiment. The main experiment consisted of 30 search trials (24 singleton-present trials and six singleton-absent trials presented in random order) and two sets of probe displays. The probe displays were introduced without any instruction. The first set of probe displays was inserted after a specific number of singleton distractor trials that differed between groups. The *critical singleton trial* refers to the search trial that immediately preceded the probe displays. There were eight *critical singleton* groups: the *Trial-1* group saw the probe trials after the first trial with a singleton; the *Trial-3* group saw probe trials after the third singleton trial. The *critical singleton trial* in each successive group occurred in increasing intervals of three trials. The last *critical singleton* group, *Trial-21*, saw the memory probe on the 21st singleton trial (see Fig. 2). These probe displays were presented without prior instruction and were, therefore, a “surprise” to the participant. All groups also experienced a second memory probe on the 24th trial as a control memory probe. However, there were no systematic differences of interest between groups, therefore the data were not included for report. Each search display remained visible until participants made a response and was followed by 200-ms of visual feedback (“correct” or “incorrect”). The trial ended with a 500-ms blank screen intertrial interval. We excluded critical search trials longer than 10-s RT from analyses. This liberal criterion was applied to only eliminate participants that were clearly disengaged from the task without unfairly excluding data from slower, more deliberate, participants. This criterion resulted in elimination of two participants.

#### Analyses

Data from each of the dependent variables were analyzed across critical singleton groups, defined by the number of singleton-present trials experienced before the *critical singleton trial* and the set of probe displays. In each case, we expected the data to show a monotonic change over groups until an asymptote was reached. For example, the RT data were expected to decrease with the number of experienced singleton distractors, indicating improved suppression, until a plateau was reached. In order to capture the expected linear change followed by a plateau, the data from each of the dependent measures were fitted using a two-part spline model. Spline models estimate multiple parameters of nonlinear data and include a “knot” point, which indicates the point at which monotonically decreasing data reaches a statistical asymptote (Cudeck & Klebe, 2002). A spline model fit with no knot point indicates that the data are fit by a single continuous line (see also Geng, DiQuattro, & Helm, 2017). To determine the best inflection point, we fit spline models with no knot point and knot points at different trial positions. The best model was determined by the one with the lowest BIC value, and model comparison was calculated with Bayes factors (BF; Wagenmakers, 2007). The BF we calculated reflects *evidence in favor of the best fitting model* (see Table 1). As a heuristic, BF values less than 3 are considered weak evidence, between 3 and 10 as moderate evidence, up to 30 as strong evidence, up to 100 as very strong evidence, and above 100 is considered extreme evidence (Stefan et al., 2019).

**Table 1:** Spline model fits for data in Experiments 1A and 1B

	Experiment 1A Search RT	Experiment 1B Search RT	Experiment 1A Color memory	Experiment 1B Color memory	Experiment 1A Awareness confidence	Experiment 1B Awareness confidence
Model 1: Linear regression	5,248.93 (BF_41_ = 7.57)	5,222.52*	1,649.24 (BF_21_ = 244.69)	1,465.56*	1,404.24 (BF_51_ = 1.34)	1,272.74*
Model 2: Knot at Trial 3	5,248.97 (BF_42_ = 7.73)	5,228.27 (BF_12_ = 17.73)	1,638.24*	1,470.54 (BF_12_ = 12.06)	1,407.664 (BF_52_ = 7.40)	1,276.34 (BF_12_ = 6.05)
Model 3: Knot at Trial 6	5,247.88 (BF_43_ = 4.48)	5,228.06 (BF_13_ = 15.96)	1,640.93 (BF_23_ = 3.84)	1,470.71 (BF_13_ = 13.13)	1,405.21 (BF_53_ = 2.18)	1,277.48 (BF_13_ = 10.70)
Model 4: Knot at Trial 9	5,244.88*	5,227.58 (BF_14_ = 12.55)	1,644.43 (BF_24_ = 22.08)	1,471.32 (BF_14_ = 17.81)	1,403.85 (BF_54_ = 1.10)	1,276.38 (BF_14_ = 6.17)
Model 5: Knot at Trial 12	5,246.42 (BF_45_ = 2.16)	5,227.97 (BF_15_ = 15.25)	1,646.72 (BF_25_ = 69.41)	1,471.29 (BF_15_ = 17.55)	1,403.66*	1,274.57 (BF_15_ = 2.50)
Model 6: Knot at Trial 15	5,247.73 (BF_46_ = 4.16)	5,228.26 (BF_16_ = 17.63)	1,649.55 (BF_26_ = 285.72)	1,471.33 (BF_16_ = 17.90)	1,405.23 (BF_56_ = 2.19)	1,274.9 (BF_16_ = 2.94)
Model 7: Knot at Trial 18	5,249.44 (BF_47_ = 9.77)	5,228.19 (BF_17_ = 17.03)	1,648.15 (BF_27_ = 141.88)	1,471.20 (BF_17_ = 16.78)	1,406.25 (BF_57_ = 3.64)	1,276.4 (BF_17_ = 6.23)

## Experiment 1B

The purpose of Experiment 1A was to test whether repeated singleton distractors lead to better attentional suppression and decrease in memory and awareness. However, since *critical singleton trials* occurring after more exposures always occurred later in the experiment, it is possible that the observed effects were due to trial order (e.g., practice effects or fatigue) and not singleton exposure. To control for this possibility, Experiment 1B was identical to Experiment 1A, except that all singleton-present trials, save the *critical singleton* trial and the last trial, were replaced with singleton-absent trials. Thus, the *critical singleton trial* was now the first singleton distractor trial in all groups, but the trial number of the *critical singleton* still differed across groups as before. If exposure to singleton distractors is necessary for learned suppression, then there should be no evidence of suppression as a function of trial sequence in this experiment. However, if the previous results were due to order effects, the results should be identical to those of Experiment 1A despite the elimination of recurring singleton distractor trials.

### Participants

An independent sample of 381 undergraduates recruited from UC Davis participated in an online experiment (testable.org) for course credit through SONA, but 61 subjects were excluded (16.0% of subjects) due to the lower than 80% of accuracy, which led to the total of 320 undergraduates (mean age = 19.6 years, *SD* = 1.9, female = 272, male = 47, other = 1, left-handed = 23). Forty participants were randomly assigned to each of eight groups. All participants had normal or corrected-to-normal vision and provided informed consent in accordance with NIH guidelines provided through the UCD Institutional Review Board.

### Stimuli, apparatus, design, and procedure

**Fig. 3:**
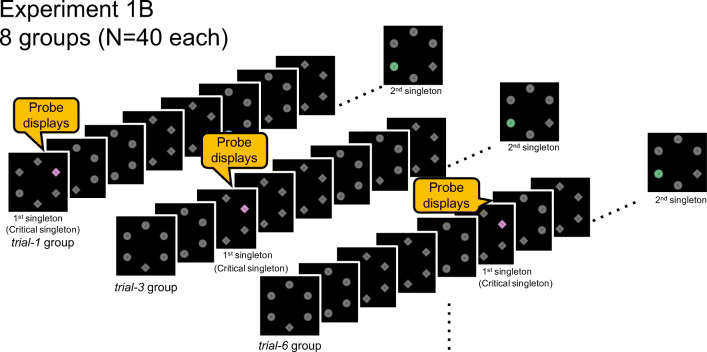
Trial procedure in Experiment 1B. All trials were singleton-absent trials except for the *critical singleton trial* and the last trial (i.e., the 2nd singleton). The trial positions of *critical singletons* and probe displays were identical with those in Experiment 1A

All aspects of Experiment 1B were identical to Experiment 1A, except that the 30 trials consisted of 28 singleton-absent trials and there was only one *critical singleton trial*. The positions of the *critical singleton trials* for eight groups were identical with those in Experiment 1A (see Fig. 3).

### Results: Experiments 1A and 1B

#### Search RTs on critical singleton trials

**Fig. 4:**
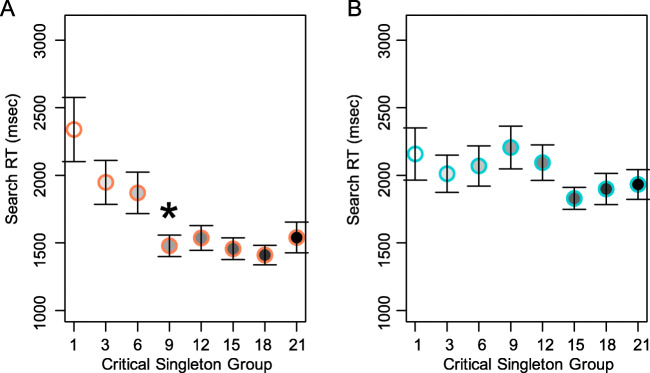
Mean search RT for the *critical singleton trial* across eight groups. **a** Experiment 1A. The “*” indicates the knot point of the best fitting model. **b** Experiment 1B. Error bars indicate ±1 standard error of the mean. *Note.* Values are model BIC and in parentheses are BF values comparing the best-fitting model to each other model. BF values indicate evidence in favor of the best fitting model. * = best fitting model

To estimate the time course of learned suppression, we analyzed search RT as a function of the *critical singleton* group (see Fig. 4) using a two-part spline model (see Methods). The spline model with the lowest BIC for Experiment 1A was Model 4, which had a knot point set at the *Trial-9* group. The model showed that attentional capture decreased linearly until about the 9th singleton repetition, after which RT reached asymptote, indicating the reduction in singleton interference reached its maximum (see Fig. 4a, Table 1). In contrast, the linear regression for data from Experiment 1B was the best fit by a model without a knot point, suggesting no consistent inflection point in performance as a function of when the singleton trial first appeared (see Fig. 4b, Table 1). The models do not include the singleton-absent trials, but performance is visualized against singleton-present trials (see Supplemental Materials, S3).

#### Color memory probe for the critical singleton

Having confirmed that singleton distractors were increasingly suppressed over repetitions, we turn next to our main research question regarding the consequence of attentional suppression on memory. We quantified memory for the color of the singleton distractor by calculating the absolute angular distance (degree) between the actual singleton color and the color wheel responses on the surprise memory probe trial (color wedges changed in 15-degree increments). Larger values indicate greater deviations from the true singleton color and, therefore, less precise memories.

**Fig. 5:**
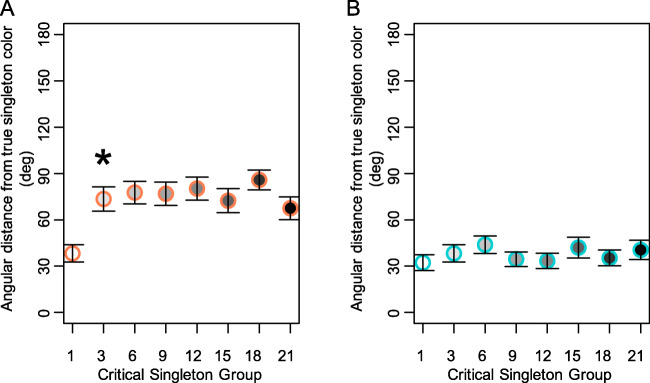
Memory for the color of the singleton distractor. **a** Experiment 1A data from color memory probes of the critical singleton distractor across eight groups. The “*” indicates the knot point of the best fitting model. **b** Experiment 1B data of color memory probes. The *critical singleton* was the first singleton encountered in all eight groups even though they appeared in a different trial position within the stream of search trials. Error bars indicate ±1 standard error of the mean

Similar to the search RT results, we fitted spline models with varying knot points to memory performance for the color memory probe trial from Experiments 1A and 1B (see Fig. 5). The results show that in Experiment 1A, Model 2 had the lowest BIC with a knot point at the *critical singleton Trial-3* group: memory error increased steeply between the first and the third singleton trials and then plateaued (see Fig. 5a, Table 1). This suggests that by the time the third singleton distractor was experienced, its color information was already filtered from memory. The fact that memory performance for the singleton distractor reached asymptote more rapidly than the search RT (i.e., by the 9th singleton) suggests partial suppression is sufficient to reduce memory for the color of singleton. In contrast, the model with no knot point was the best fit for the color memory data in Experiment 1B (see Fig. 5b, Table 1), showing that memory remained similarly precise irrespective of when the first singleton was seen within the sequence of trials.

#### Awareness and confidence ratings

Next, we assessed awareness and confidence of the color singleton’s presence. Confidence ratings were only analyzed for participants who answered “Yes” to whether or not they had seen a color singleton. First, visual inspection of the data suggests that awareness of the singleton was uniformly high in both experiments, 91.9% (*N* = 294) in Experiment 1A and 97.8% (*N* = 313) in Experiment 1B. This suggests that subjects were overall aware of the presence of singleton distractors when they occurred, although it is worth noting that responses may be biased in Experiment 1 because of the high frequency of singleton distractors (i.e., participants may have tended to guess that a singleton was present if they were not sure).

**Fig. 6:**
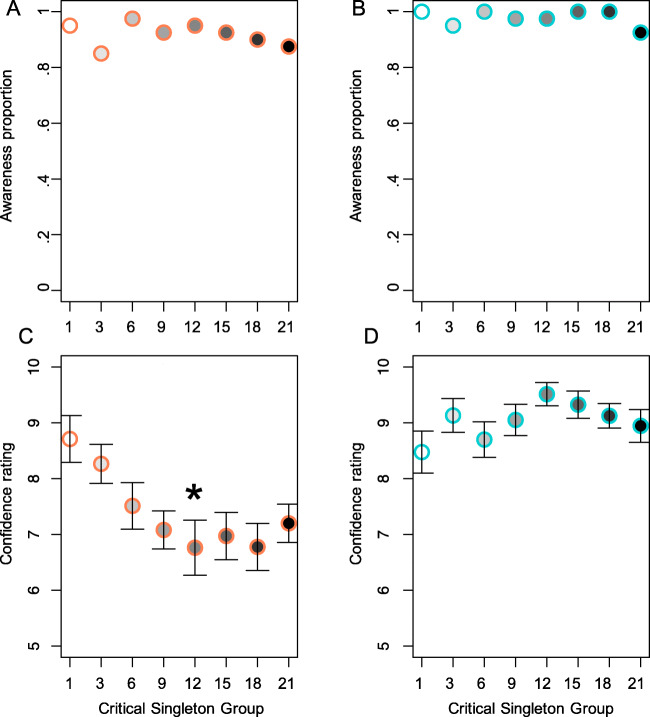
Awareness of color singleton and confidence rating. The proportion of people who said “Yes” to the question, “Was there a colored item in the previous display?” and confidence rating across eight groups. **a, c** Experiment 1A. The “*” indicates the knot point of the best fitting model. **b, d** Experiment 1B. Error bars indicate ±1 standard error of the mean.

Turning to the confidence data, the pattern was similar to RT (compare Figs. 4 and 6), showing a clear decrease in confidence that a singleton was seen on later critical singleton trials in Experiment 1A. No such pattern was seen in Experiment 1B. This observation was verified by the spline models. The best fitting model was Model 5 with a knot point at *Trial-12,* but there was only very weak evidence for this model compared with the model with a knot point at critical singleton *Trial-9* (see Table 1). This suggests that at most, by the time a color singleton was seen for the 12th time, confidence in having seen the singleton had depreciated to an asymptotic level. This pattern mirrors that of RT and suggests that as attentional suppression increased, there was a concomitant decrease in participant confidence in having seen the preceding color singleton distractor.

## Experiment 2

In Experiment 1, we found that learned suppression of salient distractors during visual search produced reduced memory performance for the color of singleton and confidence that a color singleton distractor had appeared on the previous trial. The results suggested that memory precision for the singleton declined as attentional suppression improved; however, our estimates of performance were limited by the relatively coarse color wheel and small sample size given the necessity of the one-trial surprise memory probe. In Experiment 2, we increased the sample size to 200 per group and used a more fine-grained continuous measurement of color to more precisely measure the distribution of responses for the singleton memory probe. Moreover, we chose colors that were within a single category (blue, orange) but shifted off the focal color in order to test if working memory biases towards the category center decay with learned suppression (Bae et al., 2015; Hardman et al., 2017).

### Method

#### Participants

Five hundred and seventy-seven undergraduates from UC Davis participated in Experiment 2 for course credit. We excluded 177 subjects (30.7%) due to lower than 80% accuracy or if the mouse click response was in an area outside the color and location wheels entirely. In total we had 400 participants (mean age = 20.16 years, *SD* = 2.73 years, female = 281, male = 116, other = three, left-handed = 30). The experiment was run online on the Testable platform (testable.org). Two hundred participants were randomly assigned to each of two *critical singleton* groups. All participants had normal or corrected-to-normal vision and provided informed consent in accordance with NIH guidelines provided through the UCD Institutional Review Board.

#### Apparatus and stimuli

**Fig. 7:**
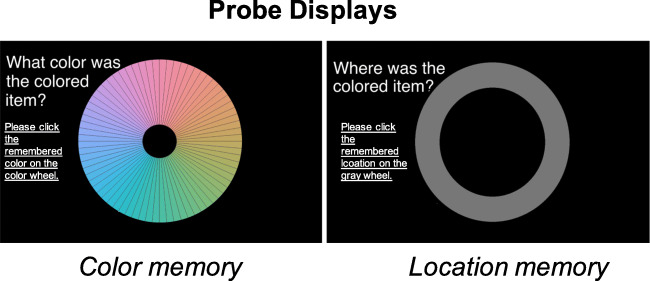
Color and location wheels used in Experiment 2. Search and probe displays were identical with those in Experiment 1. The color wheel consisted of 72 colors and the location wheel was continuous gray wheel. Participants were asked to click the color that best matched the singleton distractor and the location on the wheel that best matched the color singleton’s location on each wheel. (Color figure online)

All apparatus and stimuli were identical with Experiment 1, except that a color wheel on color memory probes now consisted of 72 colors; location memory was measured using a gray location wheel (see Fig. 7). Also, 24 equidistant singleton colors were chosen from 72 CIE Lab color wheel and we chose two critical singleton colors (RGB for the orange critical singleton: [225; 153; 118]; RGB for the blue critical singleton color: [47; 190; 194]). These colors were approximately 10 and 35 degrees from their category focal points, respectively. The distance of the blue singleton was farther because the blue category is wider (Bae et al., 2015). Both critical singleton colors were therefore between the prototypical category color and a boundary color. If a bias occurs in working memory report of the color towards the category center, the memory for the singleton color should be biased rightward (clockwise) in our measurement.

#### Design and procedure

Design and procedure were identical with Experiment 1, except that there were only two *critical singleton* groups (*Trial-1* and *Trial-12*). The *Trial-1* group saw the set of probe displays after the first singleton trial and the other group after the 12th singleton trial. The first and 12th trials were chosen because they fall on the two sides of the knot-points found in Experiment 1 such that we expected maximum capture by the first singleton and asymptotic suppression by the 12th singleton.

#### Model fitting analysis

We tested whether the precision of memory for the critical singleton differed between *critical singleton* groups by fitting response data from the memory probe trials to a “mixture model” of working memory (Zhang & Luck, 2008). The model assumes that there are two sources of error in the representation of the item in memory, and that these errors can be modeled by two separate underlying distributions: the first is a von Mises distribution, with a concentration parameter (kappa) that reflects the precision of information held in working memory; the second component is a uniform distribution which captures “guess” responses thought to result from information lost from working memory entirely. Thus, the mixture model dissociates response errors from imperfect memories and those from “guessing.”

We fit this model to the color memory probe data using custom Python code with the pymc3 package (Liew et al., 2019). We supplied priors for two free parameters in the model, the center (mu) and concentration (kappa) of the von Mises distribution. We used a prior distribution for mu that was itself a von Mises distribution with center on zero and precision of .05 (mu = 0, kappa = .05). For kappa, we assumed a uniform distribution between zero and 1,000. Kappa is bounded on the lower end by zero representing complete imprecision. The uniform distribution was set between negative pi and pi, representing an equal likelihood of all responses during guessing.

### Results

#### Critical singleton search RTs

We excluded trials with RTs over 10-s from analyses, which included data from two participants. We conducted an independent *t* test to compare RT between *critical singleton* groups. RT in the *Trial-12* group was shorter than the *Trial-1* group, *t*(294.29) = 10.35, *p* < .001, Cohen’s *d* = 1.04, BF > 100, which is a replication of the suppression effect in Experiment 1 (see Fig. 9).

#### Color memory distributions for critical singleton color

**Fig. 8:**
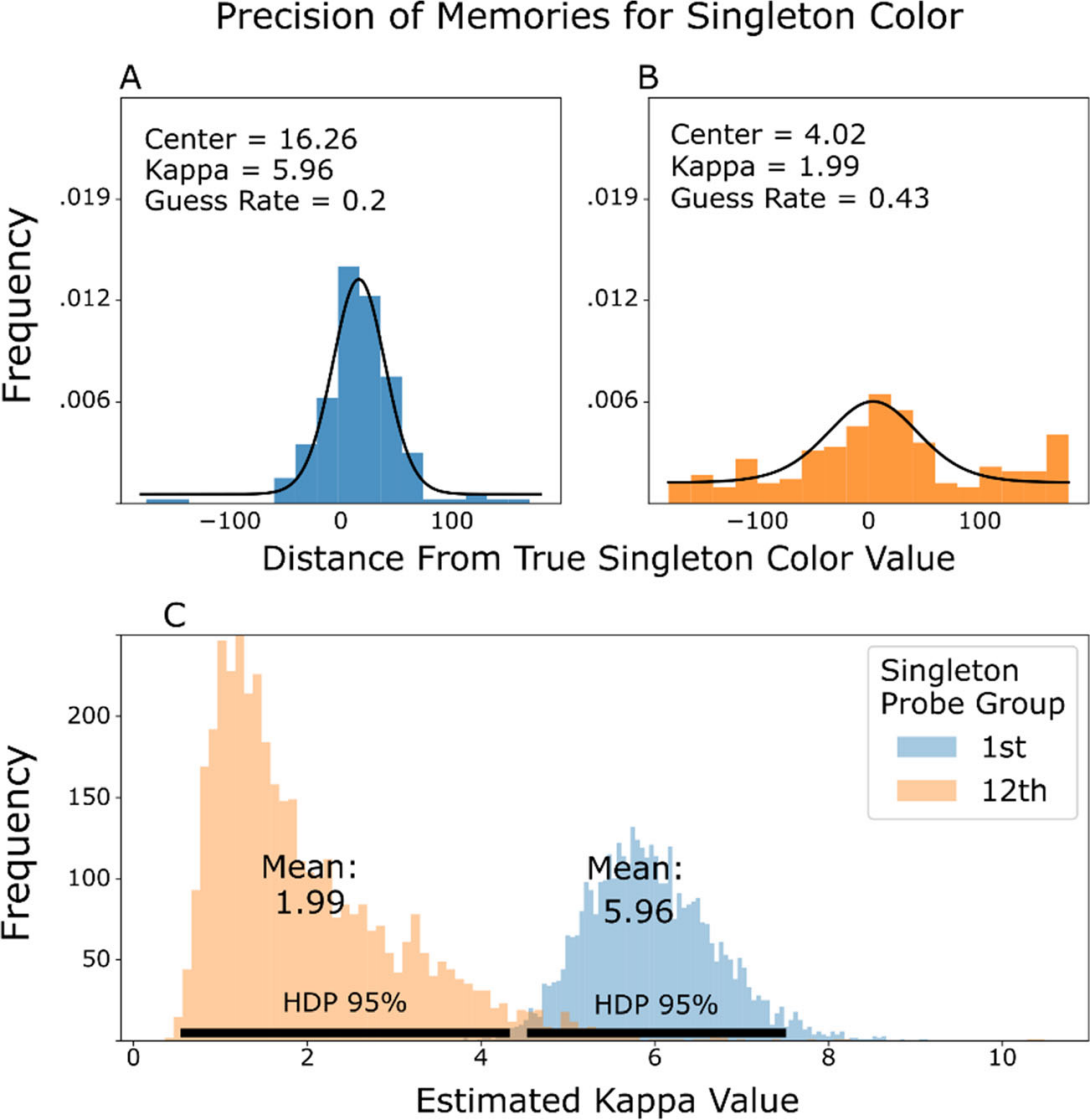
**a** Best fit working memory mixture model for the *Trial-1* group (blue), overlayed on the distribution of actual responses. Distance from the true singleton color value is in color degrees. The Center parameter reflects the central tendency of the distribution. The rightward shift is towards the color category center, suggesting a center-bias in memory. Kappa reflects the precision of the memory where larger numbers indicate greater precision. Guess rate indicates the probability that the probed item was not present in memory at the time of the probe. **b** Same as **a,** but for the *Trial-12* group (orange). **c** Distribution of posterior estimates of the concentration parameter (kappa) for each group. Black bars at the bottom indicate the 95% HDI for each group. Precision was significantly greater for the *Trial-1* group compared with the *Trial-12* group. (Color figure online)

To test whether the error in color response distribution differed between groups, we looked at the posterior distribution of the concentration parameter (kappa) estimated from fitting each model. Figure 8 shows the distribution and marks the Highest Density Interval (HDI) for each group, which describes the 95% most credible values for the parameter (Kruschke, 2018). The fitted models estimated the value of kappa to be 5.96 (HDI 95% = 4.58,7.46) for the *Trial-1* group and 1.99 (HDI 95% = 0.59, 4.28) for the *Trial-12* group. The fact that these intervals are non-overlapping suggests that the distribution for the *Trial-1* group was more precise than that of the *Trial-12* group. This indicates that while memory representations were relatively precise for the first color singleton distractor, representations of the 12th singleton distractor were poorer.

Similarly, we tested whether the guess rate differed between groups. The results showed that the guess rate estimated by the model was higher in the *Trial-12* (mean *=* 0.43, HDI 95% = 0.31, 0.56) group compared with the *Trial-1* group (mean *=* 0.2, HDI 95% = 0.15, 0.26), indicating that the *Trial-12* group was more likely to randomly guess the color of the singleton distractor.

Finally, it is worth noting that the center values of the data were both right-shifted although clearly more so for the *Trial-1* group (mean = 16.26, HDI 95% = 12.09, 20.29). Recall that colors were between the color category center (defined by Bae et al., 2015) and a color boundary. The orange and blue singleton colors were approximately 10 and 35 degrees from the category center, respectively. The shift therefore in the *Trial-1* group represents a bias to report colors closer to the category center than the true color and replicates previous research (Bae et al., 2015; Hardman et al., 2017), but this effect dissipated in the *Trial-12* group.

#### Awareness assessment and confidence ratings

**Fig. 9:**
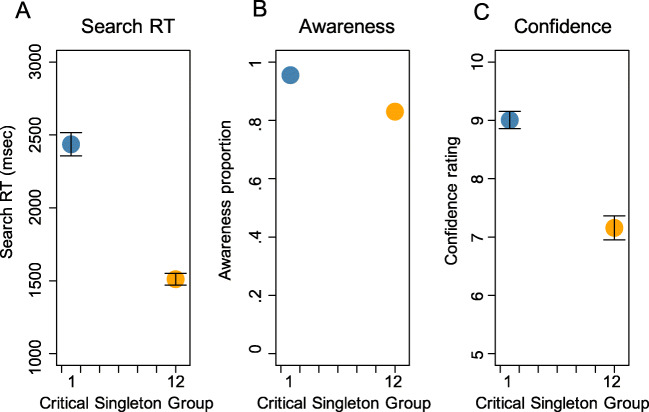
**a** Mean search RT for the *critical singleton trial* between the *Trial-1* group (blue circle) and the *Trial-12* group (orange circle)*.* **b** Awareness proportion. **c** Confidence ratings. Error bars indicate ±1 standard error of the mean. (Color figure online)

Overall awareness 95.5% for the *Trial-1 group* and 83.0% for the *Trial-12 group* (see Fig. 9) again indicating relatively high reports of awareness in both groups. The *Trial-1* group produced higher confidence in their awareness response than the *Trial-12* group, *t*(307.36) = 7.298, *p* < .001, Cohen’s *d* = .79, BF > 100. These data replicate those from Experiment 1A, confirming that while awareness was relatively high in both groups, there was an erosion of confidence in awareness when attentional suppression improved after 12 repetitions of singleton distractors.

## General discussion

There has been substantial evidence over the last decade that salient distractors can be suppressed during visual search, particularly when the distractor features recur and are predictable (Chelazzi et al., 2019; Gaspelin & Luck, 2018b; Geng et al., 2019; Noonan et al., 2018). Recent work has provided further evidence that suppression is related to participant awareness of the salient distractor (Adams & Gaspelin, 2020; Adams & Gaspelin, 2021; Constant and Liesefeld, 2021; Won et al., 2019). However, the relationship between attentional suppression of salient distractors and memory for their features and confidence in awareness has not yet been assessed. In other words, while previous studies such as Adams and Gaspelin (2020, 2021) assessed awareness of capture itself, our current study assessed the downstream cognitive consequences of capture (for early probe groups) and learned suppression (late probe groups). In two experiments, we used a “one-shot” memory probe paradigm to assess the quality of memory and awareness for a color singleton distractor on an immediately preceding visual search trial.

The visual search paradigm we used was based on Won et al. (2019) and the RT results replicated the previous pattern: Interference from a color singleton distractor declined over repetitions even though the exact color and location of the singleton could not be predicted. Furthermore, in this sample, we found that RT reached asymptote by the time the color singleton appeared for the ninth time. While we do not expect that asymptote will always be reached following a fixed number of exposures, the results suggest that learned suppression operates rapidly when the expected frequency of color singletons is high. Incidentally, we found weak evidence based on individual trial data that RTs to the color singleton were numerical shorter than the average singleton-absent trials in the *Trial-*9 and higher groups (see Supplemental Materials, S3). These results are consistent with a number of studies in the literature showing that distractor suppression improves with exposure (Awh et al., 2003; Noonan et al., 2016; Turatto et al., 2018; Vatterott et al., 2018) and learning occurs rapidly (Vatterott et al., 2018; Vatterott & Vecera, 2012).

There are several possible sources of the learned suppression in our paradigm in which the color singleton was always a distractor, and the shape singleton was always the target. This paradigm was chosen because we expected participants to use singleton search mode (Bacon & Egeth, 1994; Leber & Egeth, 2006). If so, the results could have been obtained if the color singleton was suppressed by down-weighting the color dimension entirely (Liesefeld & Muller, 2019) or by applying suppression to the strongest bottom-up visual saliency signal (Zhang et al., 2012). It is also possible that suppression was supported by a shift from singleton detection mode to feature-detection mode, for example by determining the majority shape on each trial and searching specifically for that shape; this last possibility seems less likely, however, because the target was defined by being a shape singleton on every trial and using a two-step shape strategy would be complex (Bacon & Egeth, 1994; Leber & Egeth, 2006). While the specific mechanism involved still requires more research, there is evidence from fMRI and EEG that learned suppression for distractor features operates in visual cortex, directly attenuating the attend-to-me signal and preventing attentional capture (Adam & Serences, 2020; van Moorselaar et al., 2020; Won et al., 2020). Such results suggest that learned suppression attenuates the readout of visual information about the distractor to higher order areas (Won et al., 2020) and predict that memory representations of the distractor color and awareness of the singleton should decline with increasing suppression over exposures.

The current experiments tested this relationship between suppression and memory and awareness directly by introducing a “one-shot” memory probe following visual search trials with different numbers of preceding singleton trials. Because it is impossible to ask subjects to report on distractors more than once without the distractors becoming “task-relevant,” we chose to recruit many participants and acquire responses from only one critical trial per person. Consistent with the notion that learned suppression operates on visual processing, we found that memory probe performance degraded as the number of exposures to the singleton distractor increased. Participants in later *critical singleton* groups had poorer memory representations for the color of the distractor singleton and they had lower confidence in their awareness report of the singleton being present. This finding from Experiment 1 was bolstered in Experiment 2 using the mixture model to formally estimate memory precision and guess rates: memories after the 12th singleton was significantly less precise than after the first singleton, and guess rates were significantly higher. Additionally, the expected response bias towards the category center (Bae et al., 2015; Hardman et al., 2017), was more pronounced after *Trial-1* than *Trial-12*, suggesting that the category bias is stronger when memories are relatively precise.

Although memory degraded with increased learned suppression, we found that the patterns of memory degradation and RT decreases were not identical in Experiment 1. The maximum degradation in memory from Experiment 1 occurred rapidly, reaching asymptote after just three experiences with the singleton distractor. The fact that memory performance reached asymptote so early, compared with RT declines, suggests that as soon as a visually salient object is tagged as being “task-irrelevant,” attentive processing of that stimulus is attenuated, reducing the likelihood of its feature information being stored in working memory and entering awareness.

In contrast to the decay in memory precision, which occurred early and was sustained, confidence in awareness decayed more slowly over time. The confidence results were more similar to RT, suggesting there might be a closer link between the strength of suppression and confidence in having seen a distractor at all. In these experiments, confidence appeared to track RT costs better than awareness, but the results were based on a single trial per participant and therefore may not have had elicited the range of responses possible from repeated measures (Adams & Gaspelin, 2020). Furthermore, the binary awareness question may not have been a good assessment of actual awareness from the previous trial since most trials had a color singleton and participants may have used that to “guess” that a singleton was present.

In sum, our results provide evidence that the attentional suppression operates as a gating mechanism that reduces the likelihood of task-irrelevant information being readout into memory and awareness. This “gating” function prevents further processing of distractors and appears analogous and opposite to effects of target selection in which processing of sensory features that match the target template is facilitated and pass through into memory and awareness. Future work is needed to map the exact relationship between learned suppression, memory, and awareness, but these data contribute to a better understanding of how learned suppression prevents sensory readout and affects information representations in memory and awareness.

## Supplementary Information

ESM 1 (DOCX 314 kb)
