# Supplementary material for: Memory precision for salient distractors decreases with learned suppression
Source: Psychon Bull Rev. 2021 Jul 28;29(1):169–81. doi: 10.3758/s13423-021-01968-z (PMC8815312; doi:10.3758/s13423-021-01968-z)
Supplement: Supplementary file 1 — (DOCX 314 kb) [file 13423_2021_1968_MOESM1_ESM.docx]

Supplementary Information

S1. Participant demographic information

Experiment 1A

*trial-1 group*: N=40, mean age=20.7 years, SD=3.0, 29 females, 11 males, 4 left-handed; *trial-3 group*: N=40, mean age=20.0 years, SD=2.6, 30 females, 10 males, 4 left-handed; *trial-6 group*: N=40, mean age=20.4 years, SD=1.6, 30 females, 10 males, 4 left-handed; *trial-9 group*: N=40, mean age=20.5 years, SD=1.6, 28 females, 12 males, 2 left-handed; *trial-12 group*: N=40, mean age=21.0 years, SD=2.9, 21 females, 19 males, 2 left-handed; *trial-15 group*: N=40, mean age=20.2 years, SD=2.4, 24 females, 15 males, 1 other, 5 left-handed; *trial-18 group*: N=40, mean age=20.2 years, SD=1.7, 29 females, 11 males, 2 left-handed; *trial-21* *group*: N=40, mean age=21.1 years, SD=3.0, 28 females, 11 males, 1 other, 4 left-handed.

Experiment 1B

*trial-1 group*: N=40, mean age=19.9 years, SD=1.8, 37 females, 3 males, 2 left-handed; *trial-3 group*: N=40, mean age=19.4 years, SD=1.8, 34 females, 6 males, 6 left-handed; *trial-6 group*: N=40, mean age=20.1 years, SD=3.4, 34 females, 6 males, 3 left-handed; *trial-9 group*: N=40, mean age=19.6 years, SD=1.0, 35 females, 5 males, 4 left-handed; *trial-12 group*: N=40, mean age=19.5 years, SD=1.8, 32 females, 8 males, 1 left-handed; *trial-15 group*: N=40, mean age=19.4 years, SD=1.3, 37 females, 3 males, 6 left-handed; *trial-18 group*: N=40, mean age=19.6 years, SD=1.7, 31 females, 8 males, 1 other, 0 left-handed; *trial-21 group*: N=40, mean age=19.4 years, SD=1.1, 32 females, 8 males, 1 left-handed.

Experiment 2

*trial-1 group with orange critical singleton*: N=100, mean age=20.33 years, SD=3.82, 78 females, 7 left-handed; *trial-12 group with orange critical singleton*: N=100, mean age=19.90 years, SD=1.97, 66 females, 33 males, 1 other,11 left-handed; *trial-1 group with blue critical singleton*: N=100, mean age=20.57 years, SD=2.89, 68 females, 31 males, 1 other, 10 left-handed; *trial-12 group with blue critical singleton*: N=100, mean age=19.82 years, SD=1,66, 69 females, 30 males, 1 other, 2 left-handed.

S2. Overall search accuracy and RT across groups

Table S1. Average search accuracy (proportion correct) of eight groups in Experiment 1A and Experiment 1B, and two groups in Experiment 2. The value in the parentheses is the standard deviation.

|  | *trial-1* | *trial-3* | *trial-6* | *trial-9* | *trial-12* | *trial-15* | *trial-18* | *trial-21* |
| --- | --- | --- | --- | --- | --- | --- | --- | --- |
| Exp1A | 96.3 (4.1) | 95.3 (6.5) | 96.0 (3.9) | 95.1  (4.8) | 96.3 (3.9) | 96.5 (4.5) | 96.5 (5.3) | 95.4 (5.1) |
| Exp1B | 95.3 (6.6) | 94.3 (6.5) | 94.6 (5.6) | 95.3 (6.2) | 97.1 (4.1) | 96.0 (6.1) | 95.9 (4.5) | 95.7 (4.7) |
| Exp2 | 95.9 (5.07) |  |  |  | 96.2 (4.93) |  |  |  |

(*Sub-group accuracy in Experiment 2: *trial-1 group with orange critical singleton* = 95.6%, SD = 4.8; *trial-12 group with orange critical singleton*= 96.5%, SD = 3.9; *trial-1 group with blue critical singleton* = 96.2%, SD = 5.3; *trial-12 group with blue critical singleton* = 95.9%, SD = 4.8)

Table S2. Average search RT (in ms) of eight groups in Experiment 1A and Experiment 1B, and two groups in Experiment 2. The value in the parentheses is the standard deviation.

|  | *trial-1* | *trial-3* | *trial-6* | *trial-9* | *trial-12* | *trial-15* | *trial-18* | *trial-21* |
| --- | --- | --- | --- | --- | --- | --- | --- | --- |
| Exp1A | 1798.5 (573.7) | 1813.2 (498.4) | 1920.4 (573.6) | 1649.2 (422.3) | 1807.6 (624.5) | 1613.6 (362.4) | 1785.7 (492.6) | 1704.0 (399.4) |
| Exp1B | 1822.9 (878.0) | 1659.4 (635.7) | 1720.4 (656.3) | 1625.3 (606.6) | 1511.0 (346.2) | 1504.7 (424.2) | 1547.3 (383.7) | 1343.9 (316.6) |
| Exp2 | 1840.5 (1173.7) | 1777.0 (983.9) |  |  |  |  |  |  |

(*Sub-group RTs in Experiment 2: *trial-1 group with orange critical singleton* = 1841.3, SD = 488.5; *trial-12 group with orange critical singleton* = 1821.6; SD = 590.5; *trial-1 group with blue critical singleton* = 1839.7, SD = 641.6; *trial-12 group with blue critical singleton* = 1684.9, SD = 443.7)

S3. Search RTs across all 24 distractor-present trials per trial group.

Figure S1. Search RTs across all 24 distractor-present trials with the average RT for the six distractor-absent trials for each group illustrated by the gray dotted line. The search RT for the critical singleton trial (black dot) became shorter in the later groups, consistent with a reduction in distractor interference. Following that trial, RTs increased in every group suggesting slowing related to the surprise probe trial. Although coarse, these data also show that from the trial-9 group on, the critical singleton trial RT was shorter than the average singleton-absent trial RT (dotted line) suggesting suppression beyond the distractor-absent baseline.

S4. Location memory probe performance for the critical singleton distractor in Experiment 1

Table S3. Spline model fits for location memory data in Experiments 1A and 1B. Values are model BIC. * = best fitting model

|  | Exp1A | Exp1B |
| --- | --- | --- |
| Model 1: linear regression | 1013.19 | 790.34* |
| Model 2: knot at trial 3 | 1004.26* | 795.77 |
| Model 3: knot at trial 6 | 1010.26 | 795.18 |
| Model 4: knot at trial 9 | 1012.92 | 795.09 |
| Model 5: knot at trial 12 | 1012.68 | 795.91 |
| Model 6: knot at trial 15 | 1015.14 | 796.08 |
| Model 7: knot at trial 18 | 1018.56 | 795.90 |

Figure S2. Memory for the location of the singleton distractor. Error bars indicate ±1 standard error of the mean.

S5. Location memory performance for the critical singleton distractor in Experiment 2

We followed the same procedure for fitting responses about the location of the critical singleton as we used for color responses in Experiment 2. The same priors for all parameters. We found that the location responses for *trial-1* group (Mean = 43.45, HDI= 26.11, 63.35) were more precise compared to that of *trial-12* group (Mean = .38, HDI=.04, .7). This shows that subjects in *trial-1 group* were better able to remember the location of the singleton distractor.


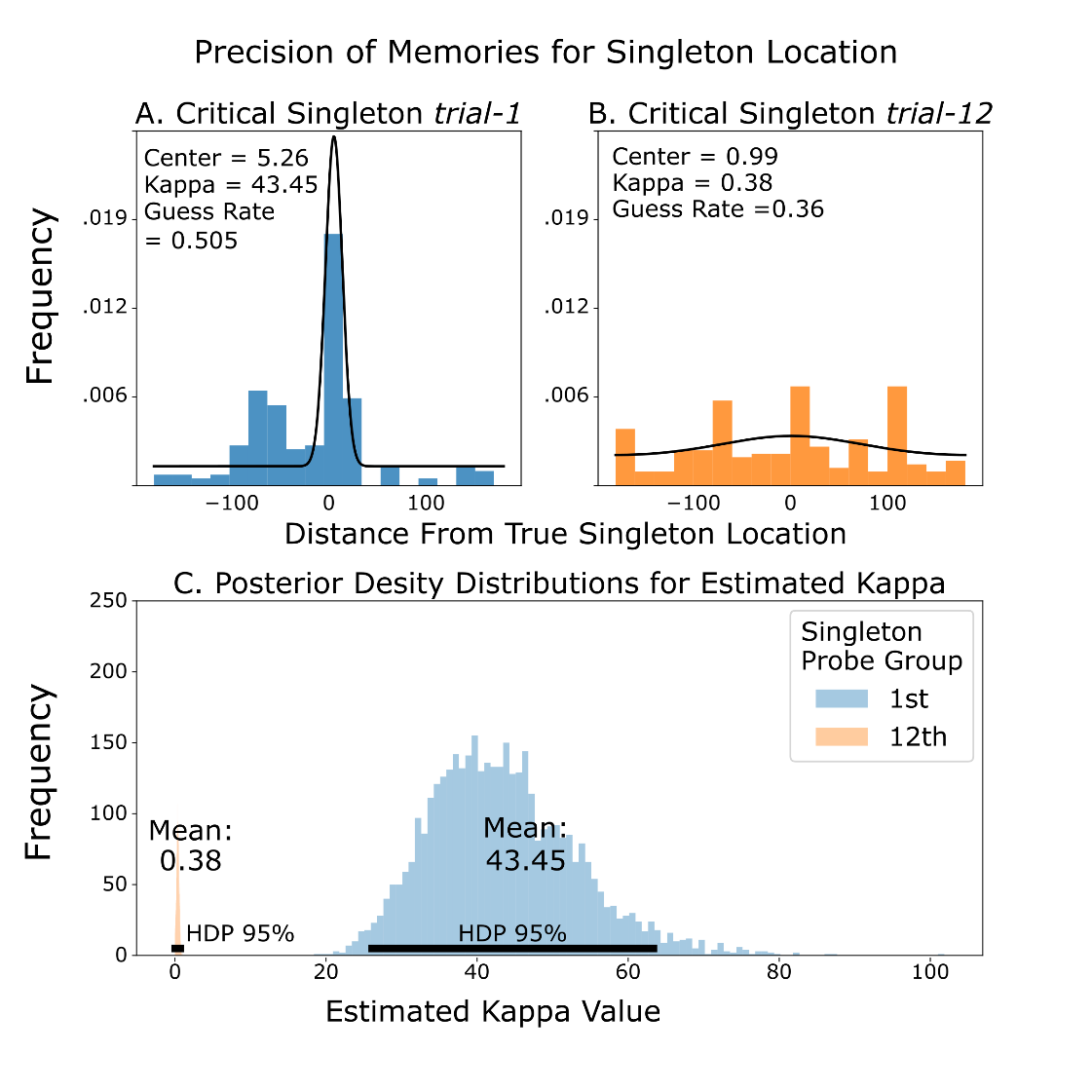


Figure S3. A. Best fit working memory mixture model for the *trial-1* group (blue), overlayed on the distribution of actual responses. Distance from the true singleton location in degrees. The Center parameter reflects the central tendency of the distribution. Kappa reflects the precision of the memory where larger numbers indicate greater precision. Guess rate indicates the probability that the probed item was not present in memory at the time of the probe. B. Same as A but for the *trial-12* group (orange). C. Distribution of posterior estimates of the concentration parameter (kappa) for each group. Black bars at the bottom indicate the 95% HDI for each group. Precision was significantly greater for the *trial-1* group compared to the *trial-12* group
